# A cortical theory of super-efficient probabilistic inference based on sparse distributed representations

**DOI:** 10.1186/1471-2202-14-S1-P324

**Published:** 2013-07-08

**Authors:** Rod Rinkus

**Affiliations:** 1 Neurithmic Systems, 275 Grove St., Suite 2-400, Newton, Mass, 02466, USA

## 

**Figure 1:**
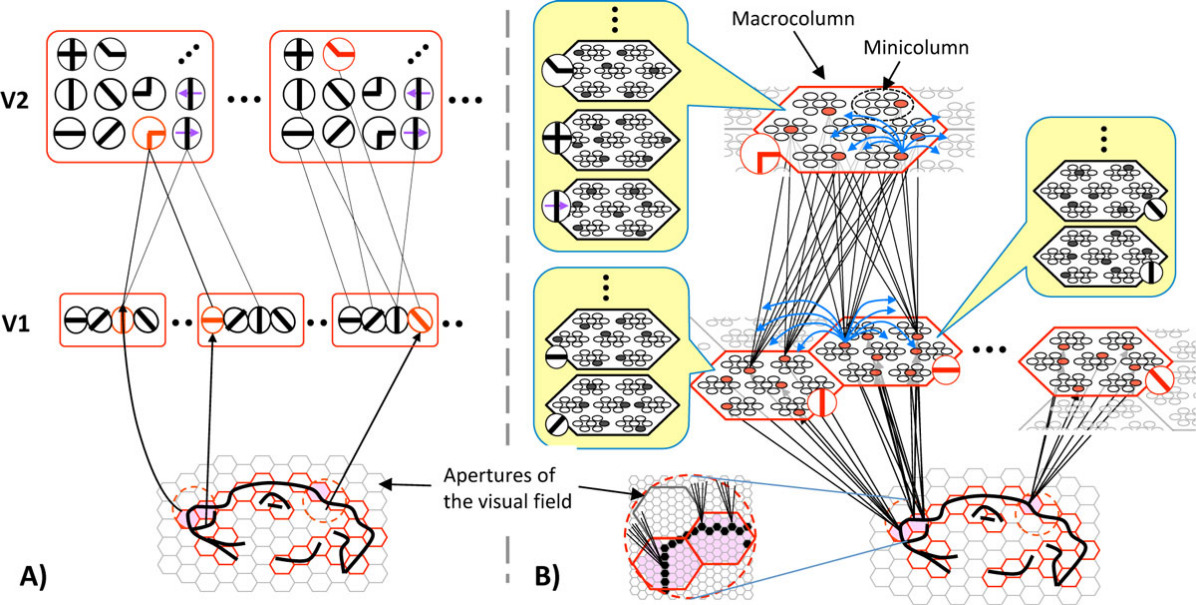
**Comparison of localist (A) and SDR-based (B) versions of visual feature hierarchies**.

The remarkable structural homogeneity of isocortex strongly suggests a canonical cortical algorithm that performs the same essential function in all regions [1]. That function is widely construed/modeled as probabilistic inference, i.e., the ability, given an input, to retrieve the best-matching memory (or, most likely hypothesis) stored in memory. In [2], I described a cortical model for which both storage (learning) of new items into memory and probabilistic inference are constant time operations, which is a level of performance not present in any other published information processing system. This efficiency depends critically on: a) representing inputs with sparse distributed representations (SDRs), i.e., relatively small sets of binary units chosen from a large pool; and on b) choosing (learning) new SDRs so that more similar inputs are mapped to more highly intersecting SDRs. The macrocolumn (specifically, its pool of L2/3 pyramidals) was proposed as the large pool, with its minicolumns acting in winner-take-all fashion, ensuring that macrocolumnar codes consist of one winner per minicolumn. Here, I present results of large hierarchical model instances, having many levels and hundreds of macrocolumns, performing: a) single-trial learning of sets of sequences derived from natural video; and b) immediate (i.e., no search) retrieval of best-matching stored sequences. Figure 1 shows the major shift in going from the localist coding scheme present in most hierarchical cortical models, e.g., [3], to SDR coding. In Figure 1A, each feature in a coding module (red rectangle) is represented by a single unit, whereas in Figure 1B, each feature in a coding module (red hexagon) is represented by a set of co-active units, one per minicolumn. Yellow call-outs show a sample suggesting the potentially large number of other features stored in a macrocolumn. This change has a potentially large impact on explaining the storage capacity of cortex, but more importantly on explaining the speed and other characteristics of probabilistic/approximate reasoning possessed by biological brains.
